# The Relationship Between Actin Cytoskeleton and Membrane Transporters in Cisplatin Resistance of Cancer Cells

**DOI:** 10.3389/fcell.2020.597835

**Published:** 2020-10-27

**Authors:** Takahiro Shimizu, Takuto Fujii, Hideki Sakai

**Affiliations:** Department of Pharmaceutical Physiology, Faculty of Pharmaceutical Sciences, University of Toyama, Toyama, Japan

**Keywords:** cisplatin resistance, actin filament, membrane transporter, anion channel, apoptosis

## Abstract

Cisplatin [*cis*-diamminedichloroplatinum (II)] is a platinum-based anticancer drug widely used for the treatment of various cancers. It forms interstrand and intrastrand cross-linking with DNA and block DNA replication, resulting in apoptosis. On the other hand, intrinsic and acquired cisplatin resistance restricts its therapeutic effects. Although some studies suggest that dramatic epigenetic alternations are involved in the resistance triggered by cisplatin, the mechanism is complicated and remains poorly understood. Recent studies reported that cytoskeletal structures regulate cisplatin sensitivity and that activities of membrane transporters contribute to the development of resistance to cisplatin. Therefore, we focus on the roles of actin filaments and membrane transporters in cisplatin-induced apoptosis. In this review, we summarize the relationship between actin cytoskeleton and membrane transporters in the cisplatin resistance of cancer cells.

## Introduction

Cisplatin [*cis*-diamminedichloroplatinum (II)], a platinum-based anticancer drug, is a widely used chemotherapeutic drug in the treatment of various cancers including testicular, bladder, prostate, ovarian, head and neck, small cell lung, non-small cell lung, esophageal, cervical, and stomach cancers (Lebwohl and Canetta, 1998; Boulikas and Vougiouka, 2004). Cisplatin enters inside cancer cells in a balance between influx and efflux through membrane transporters. The accumulated cisplatin forms intrastrand and interstrand adducts with DNA, which interferes with DNA replication and transcription. The cisplatin-triggered DNA damage activates a variety of signaling pathways such as a tumor suppressor p53 and mitogen-activated protein kinases (MAPKs), leading to apoptotic cell death (Siddik, 2003). However, the cisplatin treatment is limited in cancer therapy because cancer cells develop acquired resistance to cisplatin. The molecular mechanisms involved in the cisplatin resistance are complicated. Generally, the following three events, (1) reduced intracellular cisplatin accumulation, (2) increased DNA damage repair, and (3) inactivation of the apoptotic signaling pathways, are associated with the cisplatin resistance (Siddik, 2003; Tanida et al., 2012; Zhu et al., 2016; Lambert and Sørensen, 2018). We, therefore, begin the review by providing an overview of the cisplatin-resistant mechanism.

## Reduced Intracellular Cisplatin Accumulation in Cisplatin Resistance

**FIGURE 1:**
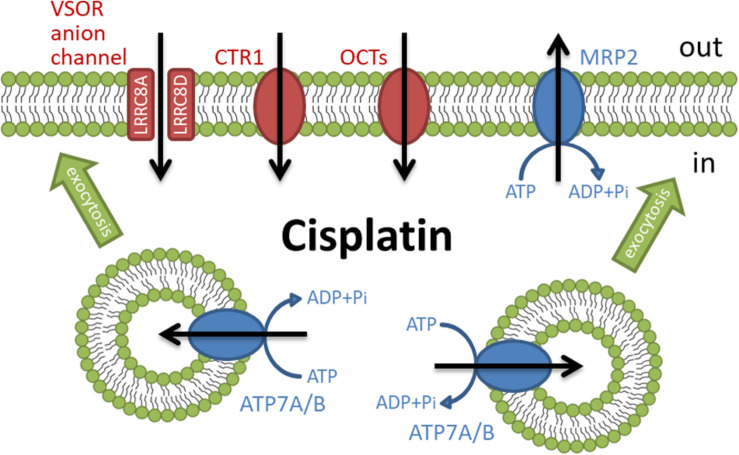
Membrane transporters involved in the regulation of cisplatin accumulation.

The independent influx and efflux pathways regulate the amount of cellular cisplatin in cancer cells (Figure 1). Although the mechanism of cisplatin uptake remains poorly understood, cisplatin passes through the plasma membrane via facilitated diffusion. The copper transporter 1 (CTR1), the first member of the solute carrier transporter 31 family (SLC31A1), is demonstrated to play a pivotal role in the cisplatin influx (Howell et al., 2010; Liu et al., 2012). The deletion of CTR1 not only reduced intracellular cisplatin accumulation but also increased the cisplatin resistance (Ishida et al., 2002; Lin et al., 2002). Besides, some cisplatin-resistant cancer cells exhibited low expression of CTR1 (Song et al., 2004; Kalayda et al., 2012). These results suggest that the downregulation of CTR1 contributes to the cisplatin resistance. The organic cation transporters (OCT), which belong to the SLC22 family, are known to transport organic cations containing medicines down an electrochemical gradient. Several reports showed that the overexpression of OCT1-3 (SLC22A1-3) enhanced cisplatin uptake and cisplatin-triggered cytotoxicity (Ciarimboli et al., 2005; Yonezawa et al., 2006; Li et al., 2012). Recently, the expression of OCT6 [SLC22A16, also called carnitine transporter 2 (CT2)] is demonstrated to contribute to the cisplatin uptake and its cytotoxicity (Kunii et al., 2015). These results suggest that these OCT proteins might function as influx transporters of cisplatin. Further studies are awaited to clarify the importance of OCT proteins in the cisplatin resistance.

Volume-sensitive outwardly rectifying (VSOR) anion channels (called volume-regulated anion channels: VRAC) are also considered to mediate cisplatin influx (Planells-Cases et al., 2015; Jentsch et al., 2016) and contribute to the cisplatin resistance (Lee et al., 2007; Shimizu et al., 2008). The VSOR anion channels generally play principal roles in cell volume recovering after cell swelling and initial cell shrinkage on apoptotic cell death (Okada et al., 2001; Shimizu et al., 2004; Pedersen et al., 2016; Okada et al., 2019). Recent studies demonstrated that the VSOR anion channels are composed of hetero-hexameric leucine-rich repeat-containing 8 (LRRC8) proteins with four membrane-spanning domains and intracellular C-terminal leucine-rich repeat domains (Qiu et al., 2014; Voss et al., 2014; Deneka et al., 2018; Kasuya et al., 2018; Kefauver et al., 2018). The LRRC8 family consists of five members LRRC8A to LRRC8E. LRRC8A is an essential component to form VSOR anion channels. Interestingly, the stoichiometry of LRRC8 proteins modifies the electrophysiological properties of VSOR anion channels. The combination of LRRC8A with LRRC8C or LRRC8E exhibits slower or faster inactivation of VSOR anion channel currents at positive potentials, respectively (Voss et al., 2014; Ullrich et al., 2016). LRRC8D regulates the permeability of VSOR anion channels (Lee et al., 2014; Planells-Cases et al., 2015). The transport of organic compounds such as osmolyte taurine, antibiotic blasticidin S, and chemotherapeutic cisplatin is dependent on the incorporation of LRRC8D into VSOR anion channels. Thus, cisplatin would pass through LRRC8D-containing VSOR anion channels. Consistently, the structural study using the cryo-electron microscopy revealed that LRRC8D homo-hexamers have a wider pore compared with LRRC8A (Nakamura et al., 2020). Intriguingly, ovarian cancer patients with low LRRC8D expression significantly exhibited poor prognosis in cisplatin therapies (Planells-Cases et al., 2015).

Some ATP-dependent active transporters are involved in the cisplatin efflux. ATP7A and ATP7B are known to be P-type ATPases to export an excess of copper (Li et al., 2018). These transporters located at the *trans*-Golgi network sequester copper from the cytosol and the accumulated copper in the *trans*-Golgi network might be released from the cell via a secretory vesicle pathway (Suzuki and Gitlin, 1999). ATP7A and ATP7B similarly transport cisplatin and regulate cisplatin sensitivity (Samimi et al., 2004). Interestingly, these transporters mainly existed at the *trans*-Golgi network in the cisplatin-sensitive cancer cells but distributed in more peripherally located vesicles in its cisplatin-resistant cells (Kalayda et al., 2008). These results suggest that cisplatin regulates the rapid trafficking of these transporters between the *trans*-Golgi network and the secretory vesicles. Moreover, several cisplatin-resistant cancer cells exhibited an increased expression of ATP7A and ATP7B (Katano et al., 2002). Multidrug resistance-associated protein 2 (MRP2), a member of the ATP-binding cassette (ABC) transporter family, also exports cisplatin as a conjugate with glutathione (Koike et al., 1997; Kawabe et al., 1999) and contributes to the cisplatin resistance (Taniguchi et al., 1996; Cui et al., 1999; Hinoshita et al., 2000). MRP2 localizes in the apical membrane of various cells and the ability of MRP2 to transport cisplatin confers the cisplatin resistance (Borst et al., 1999). Besides, cancer patients with a high level of MRP2 expression showed less sensitivity to cisplatin therapies (Korita et al., 2010; Yamasaki et al., 2011; Halon et al., 2013). Thus, the active transporters such as ATP7A/B and MRP2 regulate cisplatin efflux, although the ways to transport cisplatin are different. These results suggest that the expression of these ATP-dependent cisplatin exporters decreases intracellular cisplatin accumulation, resulting in the cisplatin resistance of cancer cells.

## Increased DNA Damage Repair in Cisplatin Resistance

Accumulated cisplatin forms interstrand and intrastrand cross-link with DNA, resulting in DNA damage. Two different pathways generally contribute to DNA repair: nucleotide excision repair (NER) and mismatch repair (MMR). The NER removes the bulky DNA adducts induced by cisplatin. On the other hand, the MMR corrects single-strand DNA errors during DNA replication. The protein expression involved in the NER and MMR processes positively and negatively correlates with the cisplatin resistance, respectively. The following reviews describe the detailed mechanism of the NER and MMR process in the cisplatin resistance (Martin et al., 2008; Rocha et al., 2018; Damia and Broggini, 2019).

## Inactivated Apoptotic Signaling Pathway in Cisplatin Resistance

Apoptosis, a programmed cell death observed in old and unwanted cells, is characterized by morphological and biochemical features such as initial cell shrinkage (called apoptotic volume decrease: AVD), cell membrane blebbing, cytochrome c release, chromatin condensation, caspase activation, DNA fragmentation, and apoptotic body formation (Maeno et al., 2000; Saraste and Pulkki, 2000; Okada et al., 2001; Barros et al., 2003). Cisplatin activates multiple signaling pathways such as reactive oxygen species (ROS), a tumor suppressor gene p53, and mitogen-activated protein kinases (MAPKs) to induce these phenomena.

As mentioned above, the VSOR anion channels mediate the cisplatin influx. On the other hand, the VSOR anion channels also contribute to the induction of AVD, a hallmark of an early stage of apoptosis. The AVD is accompanied by a coupled activation of K^+^ channels and the VSOR anion channels (Maeno et al., 2000; Okada et al., 2001; Shimizu et al., 2004). Importantly, the AVD precedes other apoptotic events because blockers of K^+^ channels and the VSOR anion channels inhibited cytochrome c release, caspase activation, and DNA fragmentation triggered by mitochondria- and death receptor-mediated apoptotic inducers in various types of cells (Maeno et al., 2000). The VSOR anion channel activities are also essential for cisplatin-induced apoptosis in human epidermoid carcinoma KB cells (Ise et al., 2005). A VSOR anion channel blocker not only suppressed caspase activation and cell death after exposure to cisplatin but also lowered the concentration dependence of cisplatin on cell viability. Intriguingly, the cisplatin-resistant cells including KCP-4 cells derived from KB cells (Lee et al., 2007), mouse Ehrlich ascites tumor cells (MDR-EATC: Poulsen et al., 2010), and human lung adenocarcinoma A549/CDDP cells (Min et al., 2011) exhibited downregulation of VSOR anion channel activities. Notably, the expression of LRRC8 members, components of the VSOR anion channel, is comparable between the parent KB cells and its cisplatin-resistant KCP-4 cells (Okada et al., 2017; Shimizu et al., 2020). These results suggest that the activation signals but not the expression of the VSOR anion channels are associated with the cisplatin resistance of KCP-4 cells. Histone deacetylases (HDACs) are essential enzymes for the regulation of gene expression. Their inhibition enhances gene transcription and reverses aberrant epigenetic changes associated with cancers (Bolden et al., 2006). Interestingly, HDAC inhibitors such as trichostatin A and apicidin recovered the function of the VSOR anion channels in KCP-4 cells, resulting in the enhanced cisplatin potency (Lee et al., 2007; Shimizu et al., 2008). This result strengthens that the AVD triggered by cisplatin-induced activation of the VSOR anion channels is pivotal for the induction of apoptosis.

We previously demonstrated that staurosporine, a mitochondria-mediated apoptotic inducer, generated ROS, resulting in the activation of the VSOR anion channels (Shimizu et al., 2004). Thus, ROS production is one of the factors inducing AVD. The mitochondrial electron transport chain in the mitochondrial inner membrane and the NADPH oxidase complex (NOX) at the plasma membrane are the major ROS generators (Liu et al., 2002; Meitzler et al., 2014; Kim et al., 2019). The mitochondrial electron transport chain generates superoxide converted to hydrogen peroxide in the intermembrane space or the matrix of mitochondria. The transmembrane enzyme NOX produces superoxide from oxygen. Cisplatin exposure induced the ROS production via the electron transport chain impairment triggered by direct damage of mitochondrial DNA (Marullo et al., 2013) and the activation of NOX isoforms at the plasma membrane (Kim et al., 2010). Some cisplatin-resistant cancer cells highly expressed superoxide dismutase 1, a superoxide scavenger, compared with the parent cells (Brown et al., 2009; Hour et al., 2010). Liu et al. (2020) recently found an increased expression of mitochondrial apurinic/apyrimidinic endonuclease 1 (mtAPE1) in cisplatin-resistant cancer cells. The mtAPE1 expression negatively correlated with intracellular ROS levels. These results suggest that the redox homeostasis contributes to the cisplatin resistance.

The activation of a tumor suppresser gene p53 is known to be essential for cisplatin-mediated apoptosis. As a transcriptional factor, p53 controls the gene transcription to promote apoptosis (Fridman and Lowe, 2003). The members of the Bcl-2 family are one of the transcriptional targets for p53. During apoptosis, p53 promotes the transcription of pro-apoptotic proteins including Bax, Puma, Noxa, and Bid (Miyashita et al., 1994; Oda et al., 2000; Nakano and Vousden, 2001; Sax et al., 2002) and suppress that of anti-apoptotic proteins Bcl-2 (Wu et al., 2001). Interestingly, cancer patients who respond to cisplatin had a higher frequency of p53-positive cells than non-responders (Garzetti et al., 1996). These suggest that the cisplatin efficacy positively correlates with the function of p53 among cancers. Importantly, half of the cancer patients carry mutations of p53 (Toledo and Wahl, 2006).

**FIGURE 2:**
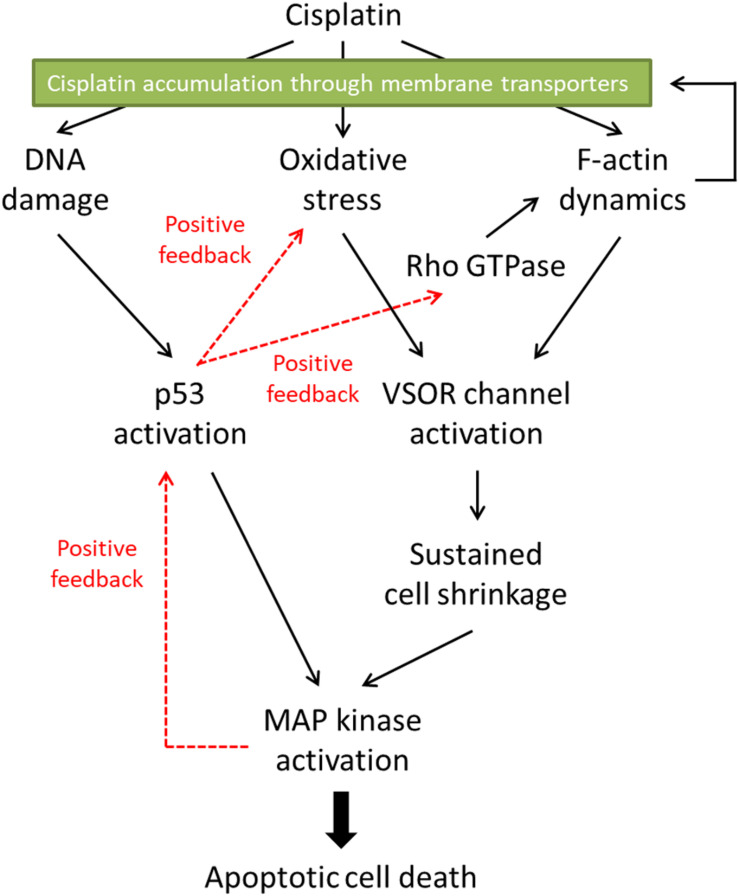
The proposed signaling pathways in cisplatin-induced apoptosis. Cisplatin-resistant cancer cells may interfere with the F-actin dynamics, resulting in the inhibition of the subsequent apoptotic signals. Furthermore, the disturbed F-actin dynamics could reduce the functional expression of cisplatin transporters on the plasma membrane, decreasing the cisplatin accumulation in the cells.

Mitogen-activated protein kinases (MAPKs), serine/threonine kinases, play pivotal roles in physiological functions such as cell survival, proliferation, migration, and apoptosis (Dhillon et al., 2007). In mammalians, members of the MAPK family include extracellular signal-regulated kinase (ERK), c-Jun N-terminal kinase (JNK), and p38 kinase. The activation of these MAPKs is essential for cisplatin-induced apoptosis. Not only cisplatin activates all ERK, JNK, and p38 kinase during apoptosis, but also reduced activation of these MAPKs correlates with the cisplatin resistance (Brozovic and Osmak, 2007). In cisplatin-induced apoptosis, the p53 transcriptional activity preceded the activation of p38 (Bragado et al., 2007). Since all MAPKs target and phosphorylate p53 at its different positions (Yue and López, 2020), on the other hand, MAPKs are an upstream signal of p53-mediated regulation. These results suggest that there might be the crosstalk between p53 and MAPKs in cisplatin-triggered signaling pathways, resulting in positive feedback loops (see Figure 2).

## The Role of the Actin Cytoskeleton in Cisplatin-Induced Apoptosis

The actin cytoskeleton is a principal structure that is essential for various cellular functions such as intracellular trafficking, contraction, motility, and apoptosis (Desouza et al., 2012). The monomer actin, a globular protein (G-actin), forms actin filaments (F-actin) by twisting two strands of G-actin. The F-actin structure is highly dynamic. The F-actin reversibly polymerizes and depolymerizes during cellular functions. The Rho family of small GTPase is an indispensable regulator of the actin cytoskeleton organization (Mokady and Meiri, 2015). The Rho GTPases function as molecular switch shifting between two conformations: a GDP-bound inactive state and a GTP-bound active state. The increased activities of Rho GTPases regulate the rearrangement of the actin cytoskeleton organization by interacting with various effector proteins.

The actin cytoskeleton dramatically changes in the apoptotic process (Desouza et al., 2012). However, the actin cytoskeleton organization during apoptosis seems to be complicated. Some cells showed actin polymerization after apoptotic stimuli (Ishimoto et al., 2011), whereas the other cells exhibited actin depolymerization during apoptosis (Udi et al., 2011; Ohno et al., 2013). Consistently, a stabilizer of F-actin, jasplakinolide, induced apoptosis (Posey and Bierer, 1999; Odaka et al., 2000). On the other hand, an inhibitor of actin polymerization, cytochalasin D, also resulted in apoptotic responses (Suria et al., 1999; Paul et al., 2002). Surprisingly, both jasplakinolide and cytochalasin D initiate apoptosis in the same human airway epithelial 1HAEo^–^ cells (White et al., 2001). These results suggest that the F-actin dynamics rather than the states of actin cytoskeleton would be associated with apoptotic induction.

**FIGURE 3:**
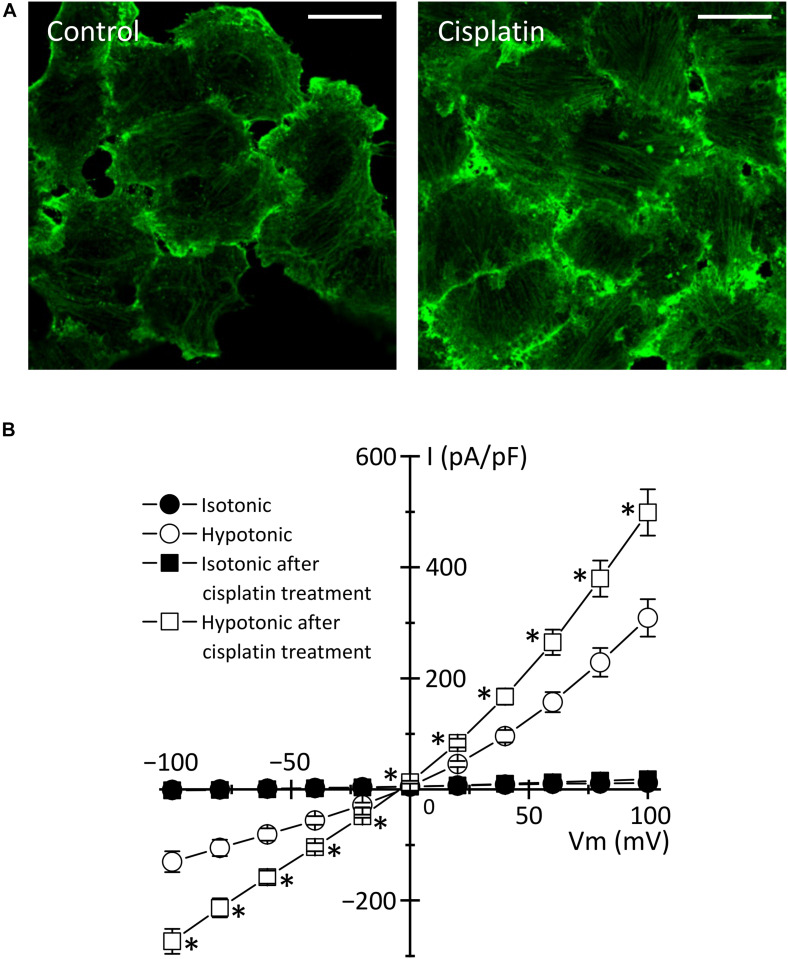
Cisplatin-triggered regulation of the actin cytoskeleton and the VSOR anion channels in human epidermoid carcinoma KB cells. KB cells were exposed to 15 μM cisplatin for 12 h. **(A)** Cellular distribution of β-actin in control (left panel) and cisplatin-pretreated (right panel) cells. Scale bars: 20 μm. **(B)** The current-voltage relationships of the VSOR anion channel currents in control (Circles: *n* = 13) and cisplatin-pretreated (Squares: *n* = 21) cells. ^∗^*P* < 0.05.

In the case of cisplatin-triggered apoptosis, the actin cytoskeleton is markedly modified. Table 1 summarizes the effects of cisplatin on F-actin in various types of cells. Cisplatin increased cell stiffness via the stabilization of F-actin in several human prostate cells (Raudenska et al., 2019) and also depolymerized F-actin in human mammary carcinoma MCF-7 cells (Zeidan et al., 2008) and porcine oocytes (Zhou et al., 2019). We observed that cisplatin enhanced the F-actin staining in KB cells (Figure 3A). Interestingly, cisplatin-induced F-actin rearrangement is reported to be an initial phase of apoptosis (Kruidering et al., 1998; Rebillard et al., 2010). Besides, cisplatin changes membrane organization and fluidity during early apoptosis (Martinho et al., 2019). Thus, the regulation of actin cytoskeleton dynamics would be a membrane-associated signaling pathway in cisplatin-induced apoptosis.

**TABLE 1:** F-actin regulation triggered by cisplatin in various types of cells.

**Cells**	**A state of the actin cytoskeleton**	**Reference**
Human prostatic epithelial PNT1A cells. Human prostate carcinoma 22Rv1 and PC-3 cells.	Increased number and length of F-actin.	Raudenska et al., 2019
Human colon carcinoma HT-29 cells	Transient actin polymerization at cell edges.	Rebillard et al., 2010
Human epidermoid carcinoma KB cells	Enhanced staining for F-actin.	Figure 3
Human mammary carcinoma MCF-7 cells	Disruption of membrane-bound F-actin.	Zeidan et al., 2008
Porcine oocytes	Impaired assembly of F-actin.	Zhou et al., 2019
Primary porcine proximal tubular cells. Porcine renal proximal tubular LLC-PK1 cells.	Depolymerization of F-actin.	Kruidering et al., 1998
Cisplatin-resistant human ovarian cancer CP70, OVCAR5-CisR, PE06, and SKOV3-CisR cells.	High density of F-actin networks.	Sharma et al., 2012, 2014
Cisplatin-resistant human epidermoid carcinoma KB-CP20 cells. Cisplatin-resistant human liver carcinoma 7404-CP20 cells.	Cluster type of actin cytoskeleton.	Shen et al., 2004
Cisplatin-resistant human epidermoid carcinoma KCP-4 cells.	Disrupted F-actin networks.	Shimizu et al., 2020

How do actin cytoskeleton dynamics modulate cisplatin-induced apoptosis? One of the answers is that F-actin regulates the expression and function of membrane transporters involved in cisplatin transport. The previous reports demonstrated that the rearrangement of F-actin increased the expression of cisplatin importer CTR1 (Abdellatef et al., 2015) and that the translocation of cisplatin exporters, ABC7A and ABC7B, from the *trans*-Golgi network to the plasma membrane is regulated by the formation of F-actin (Cobbold et al., 2002; Gupta et al., 2016). It is well known that the actin cytoskeleton regulates the VSOR anion channels, which contributes to cisplatin influx and sustained cell shrinkage during early apoptosis. The regulation patterns are dependent on cell types: the actin polymerization involves the activation of VSOR anion channels in some cells (Fatherazi et al., 1994; Zhang et al., 1997; Wei et al., 2003; Catacuzzeno et al., 2014; Burow et al., 2015), whereas the VSOR anion channel activation requires the F-actin disruption in the other cells (Levitan et al., 1995; Shen et al., 1999; Morishima et al., 2000). Intriguingly, the cisplatin-induced formation of the F-actin structure enhanced the activities of the VSOR anion channel currents in KB cells (Figure 3B). These regulations of membrane transporters by the actin cytoskeleton organization would change the cisplatin accumulation, modulating the following apoptotic processes.

## The Cisplatin-Resistant Cells Exhibit Abnormal Actin Cytoskeleton Dynamics

**FIGURE 4:**
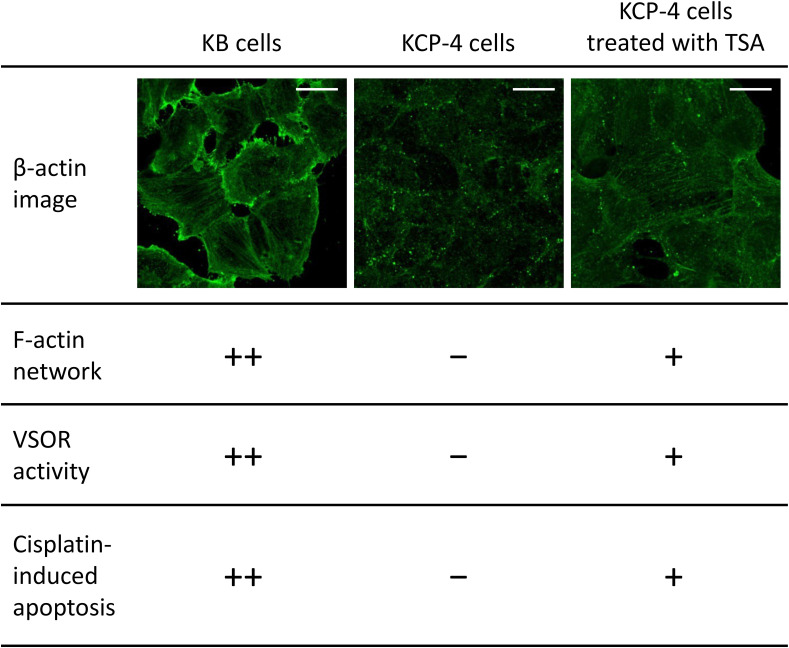
The relationship between actin network, VSOR activity, and cisplatin-induced apoptosis. Cellular distribution of β-actin in human epidermoid carcinoma KB cells **(left)**, its cisplatin-resistant KCP-4 cells **(middle)**, and KCP-4 cells treated with 400 nM trichostatin A (TSA) for 30 h **(right)** are shown in the upper lane. Scale bars: 20 μm. The degrees of F-actin network, VSOR activity, and cisplatin-induced apoptosis in each cell group are indicated as ++: strongly positive, +: positive, and –: negative. There is a close relationship between them.

Although the mechanism of the cisplatin resistance is quite complicated, the dynamic changes in the actin cytoskeleton organization are recently known to be involved in the cisplatin resistance. Some cisplatin-resistant cancer cells have higher stiffness than their parent cells sensitive to cisplatin (Sharma et al., 2012, 2014). In contrast, the other cancer cells with the cisplatin resistance exhibit the disrupted actin cytoskeleton compared with their cisplatin-sensitive cells (Figure 4; Shen et al., 2004: Shimizu et al., 2020). Although little is known about how the difference of F-actin occurs in the cisplatin-resistant cells, the different expression of Rho GTPases may contribute. The Rho subfamily of Rho GTPases composed of RhoA, RhoB, and RhoC is one of the key regulators of actin cytoskeletal organization (Aspenström et al., 2004). Cancer cells exhibit distinct expression levels of these proteins: RhoA and RhoC are highly expressed, whereas RhoB is downregulated in various human tumors (Mokady and Meiri, 2015). Interestingly, the decrease in RhoB expression was associated with the cisplatin resistance in human laryngeal carcinoma cells (Čimbora-Zovko et al., 2010). Therefore, the expression balance of these Rho GTPases might modulate the F-actin dynamics and the subsequent sensitivity to cisplatin in cancer cells. Notably, the cisplatin-induced p53 transcriptional activity is linked with the Rho GTPase pathways (Xia and Land, 2007). The epigenetic regulation of Rho GTPases by p53 may alter the F-actin organization. This pathway would cause positive feedback loops (see Figure 2), which might contribute to the chronic changes in the actin cytoskeleton of cisplatin-resistant cells. In the cisplatin resistance, the barrier function of the actin cytoskeleton might not be essential, because the cells with disturbed F-actin exhibit the cisplatin resistance. The membrane-associated signaling pathways regulated by the F-actin dynamics may modulate the cisplatin resistance.

## The Regulation of VSOR Anion Channels by Actin Cytoskeleton in Cisplatin-Resistant Cells

**FIGURE 5:**
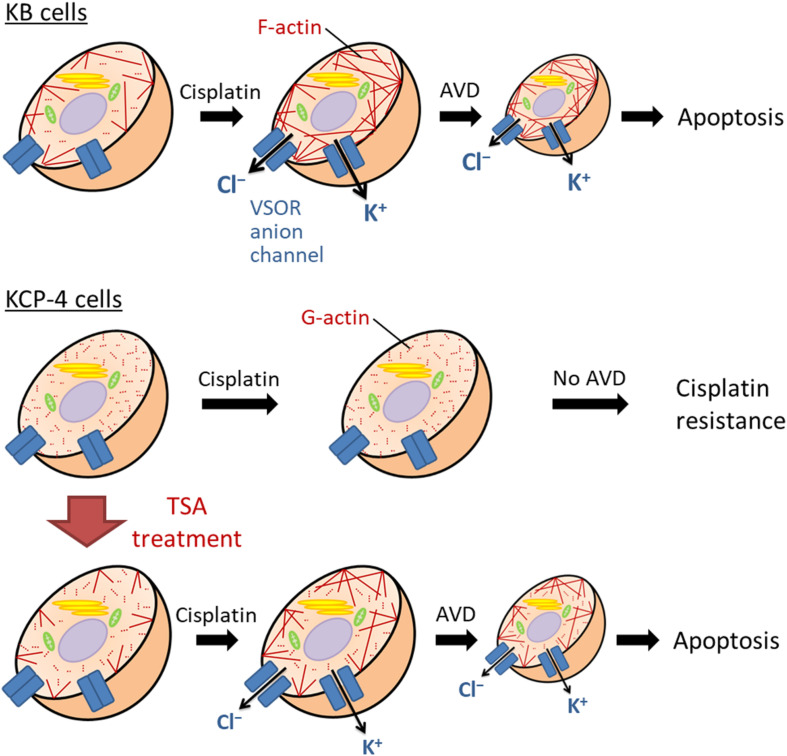
The molecular mechanism of the cisplatin resistance in human epidermoid carcinoma cells. In the cisplatin-sensitive KB cells, cisplatin induces F-actin polymerization, enhancing the activation of the VSOR anion channels and K^+^ channels. Their activation results in sustained cell shrinkage, apoptotic volume decrease (AVD), leading to apoptosis. On the other hand, the cisplatin-resistant KCP-4 cells exhibit the disruption of F-actins. The deficiency of F-actin rearrangement after cisplatin treatment attenuates the activation of the VSOR anion channels, causing the cisplatin resistance. KCP-4 cells treated with trichostatin A (TSA) shows clear F-actins, recovering the VSOR anion channel activities and its cisplatin sensitivity.

We and others previously demonstrated that some cisplatin-resistant cancer cells exhibited decreased activities of the VSOR anion channels compared with their parent cells sensitive to cisplatin (Lee et al., 2007; Poulsen et al., 2010; Min et al., 2011). In the cisplatin-resistant KCP-4 cells, interestingly, the downregulation of the VSOR anion channels was associated with the disruption of F-actin but not the expression of LRRC8 members (Shimizu et al., 2020). Additionally, the inhibition of actin cytoskeleton dynamics by β-actin knockdown or cytochalasin D treatment in the parent KB cells decreased the VSOR anion channel currents and suppressed cisplatin-induced apoptosis. Intriguingly, treatment of KCP-4 cells with an HDAC inhibitor trichostatin A, which promotes gene transcription, induced a marked increase of β-actin. The KCP-4 cells exposed to trichostatin A exhibited clear F-actin and recovered the VSOR anion channel activities, resulting in the restoration of cisplatin sensitivity (Figure 4: Lee et al., 2007; Shimizu et al., 2020). These results suggest that the defect of the VSOR anion channel activities by impaired F-actin dynamics contributes to the cisplatin resistance. Figure 5 illustrates how the VSOR anion channel modulates the sensitivity to cisplatin in KB and KCP-4 cells. As described above, the VSOR anion channels play essential roles in apoptotic induction. The dysfunction of the VSOR anion channels would decrease the cisplatin influx and suppress initial cell shrinkage during apoptosis, leading to the cisplatin resistance.

## Discussion/Conclusion

The cisplatin resistance of cancer cells is one of the therapeutic problems. In this review, we summarized the roles of the actin cytoskeleton and membrane transporters in cisplatin-induced apoptosis and the cisplatin resistance. Figure 2 indicates the proposed signaling pathways in cisplatin-induced apoptosis. Cisplatin generates oxidative stress and modulates F-actin dynamics, resulting in the activation of the VSOR anion channels. The following sustained cell shrinkage is reported to activate the stress-responsive MAPK cascade for the apoptotic induction (Hasegawa et al., 2012). Cisplatin also induces DNA damage by cross-linking or in response to generated oxidative stress (Salehi et al., 2018). The DNA damage activates a transcriptional factor p53, which causes the subsequent MAPK phosphorylation to induce apoptosis. This p53-mediated MAPK activation would be mediated by the accumulation of ROS (Shi et al., 2014). Besides, the increased activity of MAPK triggers further activation of p53 (Yue and López, 2020). On the other hand, the p53 activation would regulate the F-actin dynamics via the Rho GTPase activities (Xia and Land, 2007; Rebillard et al., 2010). These signals create positive feedback loops. Thus, the signal cascade induced by cisplatin would be complex.

Many mechanisms involved in reduced intracellular cisplatin accumulation, increased DNA damage repair, and inactivation of the apoptotic signaling pathway contribute to the cisplatin resistance. We here propose that the regulation of membrane transporters by the actin cytoskeleton dynamics has a significant role in the cisplatin resistance. The state of F-actin may not be critical, because some cisplatin-resistant cancer cells have different states of the actin cytoskeleton. The impaired F-actin dynamics may modulate the expression and function of membrane transporters carrying cisplatin. Several cisplatin-resistant cancer cells showed decreased activities of the VSOR anion channels. The actin cytoskeleton organization would contribute to the functional defect of the VSOR anion channels. However, we do not know how impaired actin dynamics modulate the VSOR anion channel activities in the cisplatin-resistant cancer cells. Further understandings of the relationship between F-actin dynamics and the VSOR anion channel function would be attractive. The VSOR anion channel is responsible for the early events of apoptosis, such as the cisplatin influx and the sustained cell shrinkage AVD. Given that reduced activities of the VSOR anion channels closely correlate with the cisplatin resistance of cancer cells, the VSOR anion channels may be one of the potential therapeutic targets to overcome the cisplatin resistance.

## Author Contributions

TS, TF, and HS wrote and edited the manuscript. All authors contributed to the article and approved the submitted version.

## Conflict of Interest

The authors declare that the research was conducted in the absence of any commercial or financial relationships that could be construed as a potential conflict of interest.
